# Malignant Struma Ovarii: A Distinctive and Rare Variant of Ovarian Tumors

**DOI:** 10.7759/cureus.104245

**Published:** 2026-02-25

**Authors:** Mohamed Kaakoua, Nada Ebbadi, Meriem Mouharir, Hamza Laabbar, Mohamed Amine Haouane, Moussa Abdoul Aziz Sawadogo, Soukayna Boujmadi, Said Kaddouri, Hassan Qacif, Abdelilah Mouhsine, Mohamed Amine Azami, Ismail Essadi, Mohamed Zyani

**Affiliations:** 1 Department of Medical Oncology, Ibn Sina Military Hospital, Marrakesh, MAR; 2 Department of Medical Imaging, Ibn Sina Military Hospital, Marrakesh, MAR; 3 Department of Internal Medicine, Ibn Sina Military Hospital, Marrakesh, MAR; 4 Department of Pathology, Ibn Sina Military Hospital, Marrakesh, MAR

**Keywords:** diagnostic approach, follicular carcinoma, ovarian tumors, rare case, struma ovarii

## Abstract

Struma ovarii is a very rare histological entity among ovarian tumors. We report the case of a 35-year-old woman presenting with pelvic pain associated with dysmenorrhea. Radiologic examination revealed a left ovarian mass with no signs of metastasis. The patient underwent surgical treatment, which confirmed the presence of thyroid tissue in the left ovary, suggestive of invasive follicular carcinoma. The aim of this case is to highlight this exceptional histological type, which first requires an appropriate diagnostic strategy to rule out metastatic involvement from a primary thyroid tumor before confirming the diagnosis of struma ovarii.

## Introduction

Struma ovarii is a rare type of ovarian tumor and was first documented by von Kalden in 1895 as a mature ovarian teratoma consisting essentially (more than 50%) or exclusively of thyroid tissue [1]. Struma ovarii represents less than 3% of all ovarian teratomas; it is a benign tumor with a risk of malignant transformation of less than 5%, referred to as “malignant struma ovarii” [2].

The clinical and radiologic data are nonspecific. Histological examination is required for the diagnosis and is based on a biopsy or surgical specimen. However, histological examination alone is not sufficient to confirm the diagnosis. Exclusion of secondary thyroid cancer localization is required. The absence of a primary thyroid tumor, according to histological examination, confirms the diagnosis [2].

In this paper, we report a case of a patient who underwent surgery for thyroid follicular carcinoma arising in struma ovarii and highlight, through this observation, the epidemiological, clinical, therapeutic, and prognostic aspects of struma ovarii.

## Case presentation

A 35-year-old woman with no relevant medical history, who was nulliparous and nulligravid, presented with left pelvic pain associated with dysmenorrhea. Clinical evaluation revealed an abdominal mass that was tender on palpation, and gynecologic examination was not possible because the patient was a virgin.

In response to this presentation, the medical team conducted a pelvic ultrasound, which confirmed the presence of a heterogeneous left adnexal mass with cystic and solid components.

**Figure 1:**
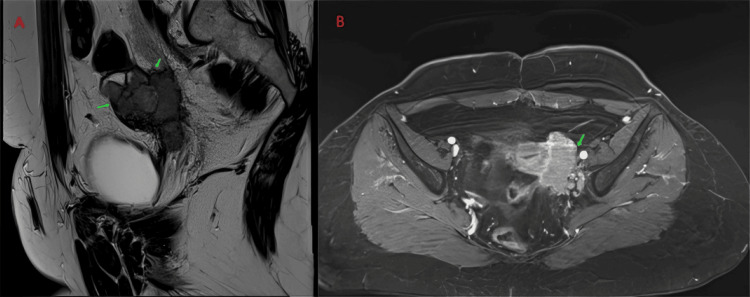
Pelvic MRI showing the presence of a solid-cystic mass in the left ovary (green arrows) (A) Sagittal section. (B) Axial section.

Pelvic MRI confirmed a solid-cystic mass originating from the left ovary, measuring 65 × 50 mm (Figure 1). The cystic component demonstrated low signal intensity on T1-weighted images and high signal intensity on T2-weighted images, whereas the solid component showed contrast enhancement after gadolinium injection. A thoracoabdominopelvic CT scan revealed no secondary lesions.

The levels of ovarian tumor markers, including cancer antigen 125 (CA125), alpha-fetoprotein, and human chorionic gonadotropin, were all within the normal range. Based on the clinical, radiologic, and biological findings, a mini-laparotomy procedure was performed. During the procedure, a cystic mass measuring 7 cm was identified, with a thickened subcapsular area containing yellowish nodules. There were no signs of effusion or peritoneal implants, and the right adnexa and uterus were intact. The surgical procedure involved a left oophorectomy, omentectomy, biopsy, and peritoneal cytology.

**Figure 2:**
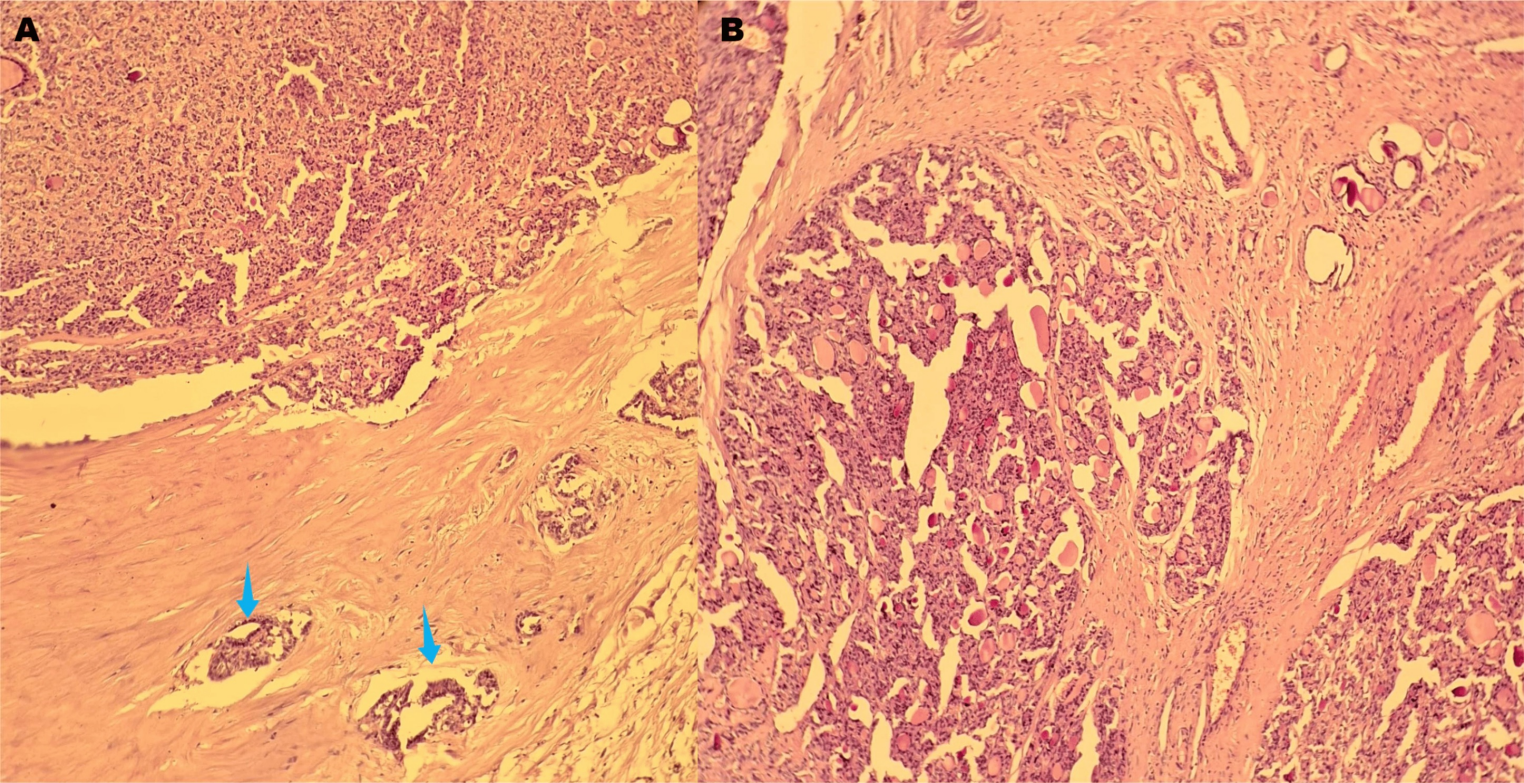
Hematoxylin and eosin-stained section showing a proliferation of thyroid-type follicles of variable size filled with eosinophilic colloid, lined by atypical cuboidal cells. The follicles are densely packed, with areas of architectural crowding and invasion, consistent with a follicular carcinoma arising in struma ovarii (ovarian goiter) (A) Low-power view (4×). (B) Higher-power view (10×). The blue arrows highlight vascular invasion, with tumor cell emboli present within vascular spaces.

On microscopic examination, the ovarian lesion was composed of thyroid-type follicles of variable size, filled with eosinophilic colloid and lined by cuboidal cells showing mild to moderate nuclear atypia. The follicles were closely packed, with loss of the normal architecture. Clear evidence of vascular and capsular invasion was observed. No papillary-type nuclear features were identified (Figure 2). Taken together, these findings support the diagnosis of a follicular carcinoma arising in struma ovarii (ovarian goiter).

The postoperative course was uneventful. Thyroid evaluation was performed and included a cervical ultrasound, which showed a normal-sized, homogeneous thyroid with no evidence of nodular lesions. The thyroid hormonal profile was normal (Table 1). Thyroid scintigraphy showed no abnormalities in thyroid uptake.

**Table 1:** Summary of biological assessment results

Test	Result	Normal values
Cancer antigen 125	16 U/mL	0-35 U/mL
α-Fetoprotein	1 ng/mL	<10 ng/mL
Human chorionic gonadotropin	2 mIU/mL	<25 mIU/mL
Thyroid-stimulating hormone	3 mIU/L	0.15-5 mIU/L
Free triiodothyronine	2.5 nmol/L	1.07-3.37 nmol/L
Thyroxine	7.5 µg/dL	5-12 µg/dL
Thyroglobulin	30 ng/mL	1.5-35 ng/mL

The multidisciplinary team recommended active surveillance. After three years of follow-up, the patient showed no signs of tumor recurrence.

## Discussion

Malignant struma ovarii (ovarian goiter) is a rare and poorly understood disease, with an incidence of approximately 0.01% among ovarian tumors [3]. It typically occurs in premenopausal women, with an average age at diagnosis of 43 years [2]. Unilateral involvement is common, while less than 6% of cases exhibit bilateral involvement [4]. Clinical presentation is variable and nonspecific, most often including lower abdominal pain, pelvic pain, or menstrual cycle abnormalities [2,5,6]. In some studies, incidental discovery was reported as the most common mode of detection of struma ovarii [5,6]. Clinical presentation with hyperthyroidism is rare, occurring in less than 8% of cases [7,8].

The ultrasound appearance of struma ovarii is nonspecific and may resemble a suspicious malignant mass with mixed solid-cystic features [9]. Pelvic MRI allows for better characterization of ovarian tumors. Struma ovarii often presents as a multilobed tumor with heterogeneous cystic areas. Cysts with hemorrhagic content show T1 hypersignal and T2 hyposignal, whereas cysts with colloid content show low signal on both T1 and T2 sequences. The solid component typically appears as a T2 hypersignal mass that enhances after gadolinium injection [10].

Biologically, the tumor marker CA125 is typically within the normal range in the absence of peritoneal effusion [11]. If the tumor secretes thyroid hormones excessively, this can result in biochemical hyperthyroidism, characterized by decreased thyroid-stimulating hormone (TSH) and elevated thyroxine and triiodothyronine. Thyroglobulin levels are often normal or elevated [4].

The most common malignant tumor arising in struma ovarii is the follicular variant of papillary carcinoma, followed by classical papillary carcinoma and then follicular carcinoma [3]. Diagnosis of malignancy is primarily histological, based on nuclear features of thyroid carcinoma and evidence of capsular and/or vascular invasion [3]. Following histological diagnosis, staging investigations are necessary to exclude ovarian metastasis from a primary thyroid tumor or possible multifocality. This typically includes thyroid ultrasound, complemented by a thoracoabdominopelvic CT scan or PET scan [3].

Treatment of localized malignant struma ovarii remains poorly standardized. Radical surgery, including hysterectomy with bilateral salpingo-oophorectomy, is considered the cornerstone. Conservative surgery, consisting of unilateral oophorectomy with complete histological staging, remains an option for women wishing to preserve fertility, as in the case of our patient [12]. The role of adjunctive therapy, such as prophylactic thyroidectomy and TSH suppression with levothyroxine or radioactive iodine, remains controversial but is recommended by some authors to reduce the risk of recurrence [13,14].

For tumors confined to the ovary, the prognosis is generally favorable and is primarily influenced by the completeness of surgical excision [14].

## Conclusions

Malignant struma ovarii is a rare and poorly understood type of ovarian tumor. Diagnosis is based on histology and the exclusion of ovarian involvement by a primary thyroid tumor. The diagnostic and therapeutic management of this rare disease should be discussed in a multidisciplinary team meeting. Surgery remains the treatment of choice, particularly for tumors confined to the ovary. The benefit of more aggressive treatment is uncertain. Overall, the prognosis is generally favorable, especially when the tumor is localized to the ovary.
